# Oral-Health-Related Quality of Life and Associated Factors in a Rural Pomeranian Population: A Cross-Sectional Study

**DOI:** 10.3390/dj14100658

**Published:** 2026-10-10

**Authors:** Lorrayne Cesario Maria, Camila Lampier Lutzke, Pamela Barbosa dos Santos, Gabriel Pereira Nunes, Maria Helena Monteiro de Barros Miotto

**Affiliations:** 1 Federal University of Espírito Santo, Vitória 29047-105, Espírito Santo, Brazil; 2 Department of Prosthodontics and Periodontology, Piracicaba Dental School, University of Campinas (UNICAMP), Piracicaba 13414-903, São Paulo, Brazil; 3 Department of Graduate Program in Public Health and Dental Science, Federal University of Espírito Santo, Vitória 29047-105, Espírito Santo, Brazil

**Keywords:** quality of life, rural population, oral health, Pomeranian population

## Abstract

**Background/Objectives:** This study estimated oral-health-related quality-of-life impacts and explored their associations with sociodemographic, dental, prosthetic, and dental-service-use characteristics among adults with direct or indirect Pomeranian ancestry living in a rural community. **Methods:** A community-based cross-sectional study included 300 adults interviewed mainly at home in the Melgaço district, Domingos Martins, Espírito Santo, Brazil, from May to August 2024. Participants were recruited through a non-probabilistic convenience approach selected to support participation in this close-knit rural setting. Recruitment was facilitated by a resident trusted by the community. The Brazilian OHIP-14 and a structured questionnaire were administered predominantly in Portuguese, with oral explanations in Pomeranian when needed. Responses of ‘often’ or ‘always’ indicated impact; a domain was considered impacted when at least one of its two items met this threshold, and overall impact was defined as at least one of the 14 items rated ‘often’ or ‘always’. Exploratory associations were assessed using central two-sided exact tests and conditional odds ratios with exact 95% confidence intervals; the analyses were unadjusted. **Results:** Overall oral health impact was reported by 68 participants (22.7%). The most frequent domain impacts were psychological discomfort (13.0%), physical pain (12.3%), and psychological disability (12.0%). Patterns of association were observed for age, dental status, perceived prosthetic need, dental-service access, and urgent dental attendance, with the precision of some estimates reflecting the small number of observations in specific cells. **Conclusions:** The findings provide an initial profile of oral-health-related quality of life in this rural Pomeranian community and identify characteristics that may be relevant to culturally responsive service planning. Within the community-based cross-sectional framework and the exploratory unadjusted analysis, the observed patterns offer a useful foundation for future adjusted and population-based investigations.

## 1. Introduction

Quality of life is defined as the sum of individual perceptions and experiences, determined by social and economic factors, personal values, and individual goals [1]. This definition highlights that quality of life transcends aspects strictly related to physical health, integrating dimensions such as autonomy, environmental conditions, and independence [1]. In the field of oral health, this approach establishes a broader perspective, incorporating the individual’s self-perception of their oral health as a complementary indicator to clinical dental assessment metrics [2]. Its applicability encompasses the formulation of more efficient strategies for the promotion and protection of collective health [3].

Several indicators have been used in current studies to analyze quality of life, among which the Oral Health Impact Profile (OHIP) stands out [4]. It is a subjective indicator, formulated in Australia in 1994 by Slade and Spencer, based on interviews with dental patients. It originally contained 49 items, categorized into seven dimensions: functional limitation, physical pain, psychological discomfort, physical disability, psychological disability, social incapacity, and impairment [4].

Reduced versions were released, notably the Oral Health Impact Profile-14 (OHIP-14), with 14 items [5]. The Brazilian version of the OHIP-14, validated in 2005 by Oliveira and Nadanovsky, presented psychometric characteristics analogous to the original English-language instrument, proving viable for differentiating groups with good and poor oral health conditions. Other factors that were validated in the measurement were the use of dental services, demand for dental treatment, and self-perception of health [6,7,8].

Pomeranian communities in Espírito Santo preserve linguistic and cultural characteristics that may shape communication with health services and perceptions of health [9,10]. Examining this community in its own context may therefore contribute perspectives that are not captured in broader rural samples, although cultural and linguistic characteristics were not evaluated as effect modifiers in the present study. Geographic distance and limited transportation may also influence access to dental care in rural areas [10].

Still in the Brazilian historical context, before the industrialization process, the country was predominantly rural [11]. With the advance of industrialization and the migratory movement towards urban areas, rural inhabitants underwent a process of marginalization, which resulted in difficulties that directly impacted their quality of life [12]. Currently, according to the 2022 demographic census [11], only about 12.6% of the population resides in rural areas. In general, Brazilian rural populations have below-average social indicators, making them vulnerable to health problems, especially when considering the social determinants that influence well-being. These individuals face significant barriers to accessing health services, such as geographical isolation and lack of transportation, which may reflect in the maintenance of low quality of life and oral health indices [12,13,14].

Previous Brazilian studies have described oral-health-related quality of life in rural populations [13,14,15], while evidence focused specifically on culturally and linguistically distinct Pomeranian communities remains limited. In particular, few studies have jointly described OHIP-14 domains, tooth loss, perceived prosthetic need, and dental-service use in this population. This study therefore estimated oral-health-related quality-of-life impacts and explored associations with sociodemographic characteristics, dental status, perceived prosthetic need, access to dental services, and reason for dental attendance among adults living in a rural Pomeranian community.

## 2. Materials and Methods

This study was approved by the Research Ethics Committee of the Health Sciences Center of the Federal University of Espírito Santo (UFES), under opinion No. 6,658,430 (Certificate of Presentation for Ethical Review No. 75250623.7.0000.5060).

A cross-sectional study was conducted in the Melgaço district, municipality of Domingos Martins, Espírito Santo, southeastern Brazil, from May to August 2024. The community is socially close-knit and preserves cultural characteristics such as the Pomeranian language and Lutheran religion [16]. Community engagement was facilitated by a resident who was known and trusted locally and who agreed to conduct the interviews. A probability-based household plan was considered; however, a community-based convenience approach was selected to support feasible and culturally acceptable participation. The interviewer invited adults from households within her local network, explained the study, and included those who were eligible and willing to participate. The final sample therefore comprised 300 adults recruited according to accessibility and willingness rather than random household selection.

Eligible participants were adults aged 18 years or older who lived in Melgaço, reported direct or indirect Pomeranian ancestry, were able to understand and answer the questionnaire, and agreed to participate. Ancestry was self-reported and supported by the community interviewer’s local knowledge; genealogical documentation was not required. The sample included participants of mixed ancestry and members of second or later generations, all of whom reported some Pomeranian ancestry. Individuals whose cognitive condition prevented comprehension of the questionnaire were not eligible.

Recruitment documentation focused on completed interviews and did not include a formal screening log. Consequently, the numbers approached, assessed for eligibility, or declining participation; the response rate; and the number enrolled per household could not be reconstructed. The analytical sample comprised 300 participants who completed the interview.

The dependent variables were the dichotomized OHIP-14 domain outcomes and the overall oral health impact outcome. Independent variables included sex, age, self-identified race/color, education, socioeconomic category, type of access to dental services, dental status, perceived need for dental prostheses, prosthesis use, and reason for dental attendance. Domain-specific analyses were considered exploratory.

Face-to-face interviews were conducted mainly in participants’ homes by one member of the Pomeranian community who was fluent in Portuguese and competent in Pomeranian. Most interviews were completed entirely in Portuguese. When a participant primarily spoke Pomeranian or requested clarification, the interviewer provided an immediate oral explanation using locally familiar Pomeranian terms. These occasional explanations supported comprehension and were distinct from a formally translated Pomeranian version of the OHIP-14; translation and back-translation, cultural adaptation, pilot testing, and equivalence assessment may be incorporated into future validation studies. Before data collection, C.L.L. provided oral guidance on questionnaire administration. Training was therefore aligned with the practical field approach, while formal calibration, ongoing supervision, and independent quality-control procedures may strengthen subsequent protocols. The Brazilian OHIP-14 and a structured questionnaire adapted from instruments used in previous rural studies were administered. The OHIP-14 contains 14 items across seven domains: functional limitation, physical pain, psychological discomfort, physical disability, psychological disability, social disability, and handicap. Each item was answered on a five-point frequency scale: never, rarely, sometimes, often, or always. Responses of ‘often’ or ‘always’ were classified as indicating impact, whereas ‘sometimes,’ ‘rarely,’ and ‘never’ were classified as no impact, consistent with the commonly used OHIP prevalence approach [5,17]. Classifiable domain and overall outcomes were available for all 300 participants. Exposure-specific analyses used available cases: missing age (n = 9) and education (n = 12) responses were excluded from the respective comparisons, and ‘did not know’ or refusal responses for dental status (n = 90) were excluded from dental-status analyses.

For each OHIP-14 domain, impact was defined as at least one of its two items being rated ‘often’ or ‘always’; overall impact was defined as at least one of the 14 items meeting this threshold. Complete dichotomized domain and overall outcomes were available for all 300 participants. The prespecified analysis focused on this prevalence-based dichotomy; continuous OHIP-14 scores and item-level missing-response patterns were outside the scope of the available analysis.

Sample-size planning assumed an estimated population of 3600 inhabitants, an expected prevalence of 35%, an absolute precision of 5 percentage points, and a 95% confidence level. Because no Pomeranian-specific prevalence estimate was available, the expected prevalence and methodological approach were based on earlier studies of rural communities conducted by the research group [18].

Sample size was estimated using the standard single-proportion formula with finite-population correction: n = N Z^2 p(1 − p)/[d^2(N − 1) + Z^2 p(1 − p)]. With N = 3600, Z = 1.96, *p* = 0.35, and d = 0.05, the estimated sample was 318.7 participants, rounded to 319. The achieved sample of 300 was close to the planned target and corresponds to a nominal precision of approximately 5.2 percentage points under simple-random-sampling assumptions. The calculation was developed for prevalence estimation and did not include a separate allowance for nonresponse or association-specific power. Because recruitment followed a community-based convenience approach, the nominal precision is best viewed as a planning parameter rather than a design-based population estimate.

Categorical variables were summarized using absolute and relative frequencies. Each independent variable was compared separately with each dichotomized OHIP-14 outcome. Overall oral health impact was the main analytical outcome, while domain-specific outcomes provided complementary exploratory information. In keeping with this exploratory objective, associations were estimated separately without multivariable adjustment. Two-by-two comparisons were examined using central two-sided exact tests (twice the smaller conditional tail probability), conditional maximum-likelihood odds ratios (ORs), and exact 95% confidence intervals (95% CIs). Calculations were performed with Python 3.12.14 and SciPy 1.17.0. No formal multiplicity adjustment was applied; domain-specific *p* values were interpreted as exploratory. Statistical significance was defined as a two-sided *p* value < 0.05.

The manuscript was prepared in accordance with the Strengthening the Reporting of Observational Studies in Epidemiology (STROBE) guideline [19].

## 3. Results

The sample included 300 participants. Most were female (57.7%), self-identified as White (73.7%), and married (65.3%). The most frequent age group was 18–29 years (28.7%), 23.3% reported 13 years of education, and 35.3% were classified in socioeconomic categories D–E. Public dental services were reported by 60.3%, 38.7% perceived a need for a dental prosthesis, and 29.3% reported urgent dental attendance. Complete baseline characteristics and missing responses are presented in Table 1.

All 300 completed interviews contributed to the overall OHIP-14 analysis. A participant-flow diagram was not constructed because screening-stage counts were not recorded. Baseline and analysis-specific denominators, including missing or excluded responses, are presented in Table 1 and the association tables.

Overall oral health impact was reported by 68 participants (22.7%). Psychological discomfort (39/300; 13.0%), physical pain (37/300; 12.3%), and psychological disability (36/300; 12.0%) were the most frequently affected domains (Table 2).

In the exact unadjusted analyses, participants aged 41 years or older showed higher odds of psychological disability than those aged up to 40 years (25/140 [17.9%] versus 10/151 [6.6%]; OR = 3.054, 95% CI 1.350–7.432; *p* = 0.005) and social disability (9/140 [6.4%] versus 2/151 [1.3%]; OR = 5.093, 95% CI 1.028–49.279; *p* = 0.045). For overall impact, the estimate suggested a possible difference but remained imprecise and was close to the prespecified significance threshold (39/140 [27.9%] versus 27/151 [17.9%]; OR = 1.770, 95% CI 0.981–3.229; *p* = 0.059). Estimates for education and race/color were likewise imprecise, with exact 95% CIs that included the null value. Sex and socioeconomic category did not show clear associations with the overall outcome (Table 3).

Dental-status analyses included 210 participants with usable responses; 90 participants who answered ‘did not know’ or declined to answer were excluded from this comparison. Physical pain was reported by 27/165 (16.4%) participants with at least one missing tooth and 1/45 (2.2%) participant with all teeth (OR = 8.557, 95% CI 1.329–360.341; *p* = 0.014). Psychological disability was reported by 29/165 (17.6%) and 2/45 (4.4%), respectively (OR = 4.561, 95% CI 1.077–41.038; *p* = 0.035). Overall impact was reported by 51/165 (30.9%) and 4/45 (8.9%), respectively (OR = 4.560, 95% CI 1.530–18.453; *p* = 0.003) (Table 4).

Psychological disability was reported by 20/116 (17.2%) participants who perceived a need for a dental prosthesis and 16/184 (8.7%) who did not (OR = 2.182, 95% CI 1.020–4.737; *p* = 0.044). Overall impact was reported by 36/116 (31.0%) and 32/184 (17.4%), respectively (OR = 2.132, 95% CI 1.190–3.837; *p* = 0.010) (Table 5).

Psychological discomfort was reported by 32/198 (16.2%) participants who used public dental services or lacked access and 7/102 (6.9%) who used private services or health insurance (OR = 2.609, 95% CI 1.077–7.280; *p* = 0.031) (Table 6). Other domain estimates for dental-service access were based on small cells or had exact 95% CIs that included the null value.

Compared with routine or preventive attendance, urgent dental attendance was associated with physical pain (21/88 [23.9%] versus 16/212 [7.5%]; OR = 3.819, 95% CI 1.783–8.334; *p* < 0.001), psychological discomfort (22/88 [25.0%] versus 17/212 [8.0%]; OR = 3.803, 95% CI 1.806–8.142; *p* < 0.001), physical disability (10/88 [11.4%] versus 3/212 [1.4%]; OR = 8.857, 95% CI 2.205–51.378; *p* < 0.001), psychological disability (18/88 [20.5%] versus 18/212 [8.5%]; OR = 2.761, 95% CI 1.276–5.983; *p* = 0.009), handicap (7/88 [8.0%] versus 1/212 [0.5%]; OR = 18.053, 95% CI 2.263–825.556; *p* = 0.002), and overall impact (36/88 [40.9%] versus 32/212 [15.1%]; OR = 3.874, 95% CI 2.119–7.137; *p* < 0.001) (Table 7).

Consistent with the exploratory objective, all reported ORs were unadjusted. Some domain estimates arose from small cell counts and correspondingly wide 95% CIs; interpretation therefore emphasizes the overall pattern and consistency of the findings rather than isolated differences in magnitude.

## 4. Discussion

This study provides an initial community-based profile of oral-health-related quality of life among adults with Pomeranian ancestry living in rural Melgaço. Overall impact was reported by 68 of 300 participants (22.7%), and psychological discomfort, physical pain, and psychological disability were the most frequently affected OHIP-14 domains. The exact analyses highlighted exploratory patterns involving age, dental status, perceived prosthetic need, dental-service access, and urgent dental attendance. These cross-sectional unadjusted estimates complement the descriptive profile and identify relationships that may be examined further in adjusted studies [20,21].

Cultural studies describe meaningful links among work, land, and perceptions of health in Pomeranian communities [22,23]. Although these perspectives help situate the present findings, the questionnaire did not directly assess cultural beliefs, attitudes toward body care, or work identity. These dimensions may be particularly valuable to investigate in future culturally informed or mixed-methods studies.

Older age was associated with psychological and social disability in the exact analyses, while the estimate for overall impact was close to the significance threshold and had limited precision. Related age patterns have been reported in studies of oral-health-related quality of life [17,20,24,25,26]. Because age may share pathways with dental status, socioeconomic conditions, and use of services, future adjusted studies can further clarify its specific contribution [24,27].

Education may influence access to health information and recognition of dental-care needs [28,29]. In the present sample, point estimates generally suggested greater impact among participants with fewer years of education, although the exact 95% CIs included the null value. This pattern provides a relevant direction for larger studies that can account simultaneously for related social and dental characteristics.

For non-White self-identification, the point estimate in the handicap domain was based on eight participants with impact and consequently had a wide exact 95% CI. Racial inequalities in access to information and health services may contribute to oral health differences [30,31], although these pathways were not directly measured. The observed pattern therefore provides a hypothesis for future investigation.

Tooth loss and perceived need for a dental prosthesis were associated with several oral health impact outcomes in the unadjusted analyses. Tooth loss may affect chewing, speech, appearance, self-esteem, and social interaction [32,33,34,35], while perceived prosthetic need may reflect functional or psychosocial consequences of missing teeth [20,33,34,35,36]. The consistency of these patterns supports attention to restorative and rehabilitative needs, while their interrelationships should be examined in future adjusted models.

Earlier research among Pomeranian descendants in Domingos Martins described early tooth loss, long periods of prosthesis use, and dissatisfaction related to chewing, appearance, and prosthesis age [37]. These observations complement the present findings on perceived prosthetic need, while differences in sampling, measurement, and study period should be considered when comparing the studies.

Participants who used public dental services or reported no access had a higher prevalence of psychological discomfort than those using private services or health insurance. Social and territorial inequalities in dental-service use persist in Brazil [38,39], including in rural communities [14]. Because service type may also reflect socioeconomic position, dental need, geographic access, and attendance patterns, this association may be understood as a marker of combined access and need rather than an effect attributable to the public service itself.

Urgent dental attendance showed the most consistent associations with OHIP-14 outcomes [40,41]. This pattern is clinically plausible because pain and acute oral problems often prompt urgent visits [40,41]. Within the cross-sectional assessment, urgent attendance and perceived impact were recorded at the same stage; longitudinal research may further clarify their temporal relationship. Transportation barriers and delayed care may also be relevant in rural settings and could be incorporated into future investigations [40,42].

Sex and socioeconomic category did not show clear associations with the overall outcome, and no statistically clear domain-specific pattern was identified for sex. The socioeconomic comparison included relatively few participants in categories A/B and some zero cells, which is relevant when considering the precision of these estimates. Future studies that directly assess work roles, gender-related health perceptions, and culturally specific experiences may add depth to these findings.

Several methodological features help define the scope of the findings. Community-based convenience recruitment and the participation of a trusted local interviewer made data collection feasible and culturally respectful in a population with limited openness to outside researchers, enabling the inclusion of 300 adults. As recruitment was not probability-based, the results most directly describe the participating group, and older residents or people with more direct Pomeranian ancestry may be less represented. Screening totals and household identifiers were not recorded, so the response rate and household clustering were not evaluated. Oral guidance for the interviewer and occasional explanations in Pomeranian supported questionnaire administration; future protocols may extend these procedures through formal calibration, documented quality control, and a culturally validated Pomeranian version of the OHIP-14. The dichotomous OHIP-14 approach provided an interpretable prevalence measure, while continuous scores and the full ordinal response range were not explored. Differences in available-case denominators, particularly for dental status, and the use of separate unadjusted comparisons without multiplicity correction support an exploratory reading of individual estimates. The cross-sectional design describes associations at one point in time. Taken together, these features do not reduce the contribution of the study as an initial community profile; rather, they establish its interpretive boundaries and provide a clear basis for more representative, linguistically validated, and analytically adjusted research in rural Pomeranian communities.

## 5. Conclusions

This study provides a community-specific profile of oral-health-related quality of life among 300 adults in rural Melgaço. Overall impact was reported by 68 participants (22.7%), most often involving psychological discomfort, physical pain, and psychological disability. The exploratory associations observed for age, dental status, perceived prosthetic need, dental-service access, and urgent attendance indicate potentially relevant priorities for culturally responsive oral health care. Interpreted within the cross-sectional, unadjusted analytical framework, these findings offer a useful foundation for service planning and for future population-based studies with culturally validated measures and adjusted analyses.

## Figures and Tables

**Table 1:** Characteristics of adults from the rural Pomeranian community included in the study, 2024.

Characteristic	n	%
**Sex**		
Male	127	42.3
Female	173	57.7
**Age**		
18–29 years	86	28.7
30–40 years	65	21.7
41–49 years	70	23.3
50 years or older	70	23.3
Missing	9	3.0
**Self-identified race/color**		
White	221	73.7
Black	17	5.7
Mixed race	60	20.0
Asian	2	0.6
**Marital status**		
Single	56	18.7
Married	196	65.3
Stable union	31	10.3
Separated	3	1.0
Divorced	8	2.7
Widowed	6	2.0
**Years of education**		
0–2 years	27	9.0
3–6 years	54	18.0
7–10 years	45	15.0
11–12 years	30	10.0
13 years	70	23.3
14–16 years	15	5.0
>16 years	47	15.7
Missing	12	4.0
**Socioeconomic category**		
A	3	1.0
B1	7	2.3
B2	28	9.3
C1	71	23.8
C2	85	28.3
D–E	106	35.3
**Access to dental services**		
No access	17	5.7
Public service	181	60.3
Private service	97	32.3
Health insurance	5	1.7
**Dentition status**		
All teeth present	45	15.0
Completely edentulous	24	8.0
One missing tooth	67	22.3
>1 missing tooth	74	24.7
Did not know	44	14.7
Declined to answer	46	15.3
**Perceived need for a dental prosthesis**		
Yes	116	38.7
No	184	61.3
**Reason for the most recent dental visit**		
Urgent care	88	29.3
Routine care	130	43.4
Prevention	82	27.3

Values are n (%) unless otherwise stated. Percentages use the total sample (N = 300).

**Table 2:** Frequency of oral-health-related quality-of-life impacts among adults from the rural Pomeranian community, 2024.

OHIP-14 Outcome	With Impact n (%)	Without Impact n (%)
Functional limitation	12 (4.0)	288 (96.0)
Physical pain	37 (12.3)	263 (87.7)
Psychological discomfort	39 (13.0)	261 (87.0)
Physical disability	13 (4.3)	287 (95.7)
Psychological disability	36 (12.0)	264 (88.0)
Social disability	11 (3.7)	289 (96.3)
Handicap	8 (2.7)	292 (97.3)
Overall impact	68 (22.7)	232 (77.3)

All OHIP-14 outcome denominators were N = 300.

**Table 3:** Exploratory unadjusted associations between sociodemographic factors and OHIP-14 outcomes, 2024.

OHIP-14 Outcome	Reference Group n/N (%)	Comparison Group n/N (%)	Unadjusted OR (95% CI)	*p* Value
**Male (reference, n = 127) versus Female (n = 173); missing n = 0**				
Functional limitation	5/127 (3.9)	7/173 (4.0)	1.029 (0.274–4.214)	1.000
Physical pain	14/127 (11.0)	23/173 (13.3)	1.237 (0.580–2.723)	0.684
Psychological discomfort	11/127 (8.7)	28/173 (16.2)	2.032 (0.933–4.724)	0.078
Physical disability	5/127 (3.9)	8/173 (4.6)	1.182 (0.332–4.711)	1.000
Psychological disability	10/127 (7.9)	26/173 (15.0)	2.065 (0.918–5.000)	0.085
Social disability	4/127 (3.1)	7/173 (4.0)	1.296 (0.321–6.172)	0.935
Handicap	3/127 (2.4)	5/173 (2.9)	1.229 (0.234–8.064)	1.000
Overall impact	26/127 (20.5)	42/173 (24.3)	1.245 (0.693–2.266)	0.525
**Up to 40 years (reference, n = 151) versus 41 years or older (n = 140); missing n = 9**				
Functional limitation	6/151 (4.0)	6/140 (4.3)	1.082 (0.282–4.154)	1.000
Physical pain	16/151 (10.6)	19/140 (13.6)	1.324 (0.614–2.888)	0.549
Psychological discomfort	17/151 (11.3)	22/140 (15.7)	1.468 (0.706–3.098)	0.346
Physical disability	3/151 (2.0)	9/140 (6.4)	3.376 (0.820–19.793)	0.105
Psychological disability	10/151 (6.6)	25/140 (17.9)	3.054 (1.350–7.432)	0.005
Social disability	2/151 (1.3)	9/140 (6.4)	5.093 (1.028–49.279)	0.045
Handicap	2/151 (1.3)	6/140 (4.3)	3.323 (0.582–34.210)	0.236
Overall impact	27/151 (17.9)	39/140 (27.9)	1.770 (0.981–3.229)	0.059
**White (reference, n = 221) versus Non-White (n = 79); missing n = 0**				
Functional limitation	8/221 (3.6)	4/79 (5.1)	1.418 (0.304–5.480)	0.784
Physical pain	23/221 (10.4)	14/79 (17.7)	1.850 (0.829–4.009)	0.140
Psychological discomfort	25/221 (11.3)	14/79 (17.7)	1.685 (0.762–3.607)	0.212
Physical disability	9/221 (4.1)	4/79 (5.1)	1.255 (0.274–4.662)	0.923
Psychological disability	24/221 (10.9)	12/79 (15.2)	1.468 (0.632–3.254)	0.411
Social disability	8/221 (3.6)	3/79 (3.8)	1.051 (0.175–4.523)	1.000
Handicap	3/221 (1.4)	5/79 (6.3)	4.878 (0.924–32.171)	0.064
Overall impact	46/221 (20.8)	22/79 (27.8)	1.466 (0.771–2.737)	0.262
**13 years or more (reference, n = 132) versus Up to 12 years (n = 156); missing n = 12**				
Functional limitation	4/132 (3.0)	7/156 (4.5)	1.501 (0.372–7.156)	0.746
Physical pain	14/132 (10.6)	20/156 (12.8)	1.239 (0.566–2.777)	0.694
Psychological discomfort	14/132 (10.6)	24/156 (15.4)	1.530 (0.721–3.358)	0.308
Physical disability	3/132 (2.3)	10/156 (6.4)	2.935 (0.735–16.954)	0.157
Psychological disability	10/132 (7.6)	24/156 (15.4)	2.212 (0.972–5.406)	0.060
Social disability	4/132 (3.0)	6/156 (3.8)	1.279 (0.296–6.301)	0.964
Handicap	2/132 (1.5)	6/156 (3.8)	2.592 (0.453–26.691)	0.405
Overall impact	22/132 (16.7)	40/156 (25.6)	1.721 (0.931–3.248)	0.087
**Socioeconomic categories A/B (reference, n = 38) versus Categories C/D/E (n = 262); missing n = 0**				
Functional limitation	0/38 (0.0)	12/262 (4.6)	Not estimable	0.381
Physical pain	1/38 (2.6)	36/262 (13.7)	5.873 (0.927–245.549)	0.068
Psychological discomfort	6/38 (15.8)	33/262 (12.6)	0.769 (0.287–2.423)	0.740
Physical disability	1/38 (2.6)	12/262 (4.6)	1.773 (0.248–77.944)	0.984
Psychological disability	4/38 (10.5)	32/262 (12.2)	1.182 (0.383–4.886)	1.000
Social disability	1/38 (2.6)	10/262 (3.8)	1.467 (0.198–65.418)	1.000
Handicap	0/38 (0.0)	8/262 (3.1)	Not estimable	0.668
Overall impact	6/38 (15.8)	62/262 (23.7)	1.651 (0.640–5.056)	0.385

Values are n/N (%). ORs compare the comparison group with the reference group. Missing data: age, n = 9; education, n = 12. OR, odds ratio; CI, confidence interval. Exact two-sided *p* values are shown. ‘Not estimable’ indicates a zero cell.

**Table 4:** Exploratory unadjusted associations between dental status and OHIP-14 outcomes, 2024.

OHIP-14 Outcome	All Teeth (n = 45) n/N (%)	At Least One Missing Tooth (n = 165) n/N (%)	Unadjusted OR (95% CI)	*p* Value
Functional limitation	2/45 (4.4)	7/165 (4.2)	0.953 (0.173–9.724)	1.000
Physical pain	1/45 (2.2)	27/165 (16.4)	8.557 (1.329–360.341)	0.014
Psychological discomfort	3/45 (6.7)	31/165 (18.8)	3.225 (0.932–17.311)	0.070
Physical disability	0/45 (0.0)	12/165 (7.3)	Not estimable	0.101
Psychological disability	2/45 (4.4)	29/165 (17.6)	4.561 (1.077–41.038)	0.035
Social disability	1/45 (2.2)	7/165 (4.2)	1.944 (0.239–89.825)	0.917
Handicap	1/45 (2.2)	6/165 (3.6)	1.657 (0.193–78.085)	1.000
Overall impact	4/45 (8.9)	51/165 (30.9)	4.560 (1.530–18.453)	0.003

Values are n/N (%). ORs compare the second group with the first. Missing dental-status data: n = 90. OR, odds ratio; CI, confidence interval. Exact two-sided *p* values are shown. ‘Not estimable’ indicates a zero cell.

**Table 5:** Exploratory unadjusted associations between perceived need for a dental prosthesis and OHIP-14 outcomes, 2024.

OHIP-14 Outcome	No Perceived Need (n = 184) n/N (%)	Perceived Need (n = 116) n/N (%)	Unadjusted OR (95% CI)	*p* Value
Functional limitation	7/184 (3.8)	5/116 (4.3)	1.138 (0.278–4.284)	1.000
Physical pain	18/184 (9.8)	19/116 (16.4)	1.803 (0.850–3.837)	0.133
Psychological discomfort	19/184 (10.3)	20/116 (17.2)	1.805 (0.867–3.774)	0.122
Physical disability	6/184 (3.3)	7/116 (6.0)	1.901 (0.532–7.039)	0.389
Psychological disability	16/184 (8.7)	20/116 (17.2)	2.182 (1.020–4.737)	0.044
Social disability	6/184 (3.3)	5/116 (4.3)	1.335 (0.314–5.389)	0.860
Handicap	4/184 (2.2)	4/116 (3.4)	1.605 (0.293–8.797)	0.748
Overall impact	32/184 (17.4)	36/116 (31.0)	2.132 (1.190–3.837)	0.010

Values are n/N (%). ORs compare the second group with the first. No missing data. OR, odds ratio; CI, confidence interval. Exact two-sided *p* values are shown. ‘Not estimable’ indicates a zero cell.

**Table 6:** Exploratory unadjusted associations between access to dental services and OHIP-14 outcomes, 2024.

OHIP-14 Outcome	Private/Insurance (n = 102) n/N (%)	No Access/Public Service (n = 198) n/N (%)	Unadjusted OR (95% CI)	*p* Value
Functional limitation	1/102 (1.0)	11/198 (5.6)	5.917 (0.838–258.214)	0.092
Physical pain	8/102 (7.8)	29/198 (14.6)	2.012 (0.854–5.305)	0.124
Psychological discomfort	7/102 (6.9)	32/198 (16.2)	2.609 (1.077–7.280)	0.031
Physical disability	1/102 (1.0)	12/198 (6.1)	6.489 (0.935–281.258)	0.064
Psychological disability	8/102 (7.8)	28/198 (14.1)	1.931 (0.816–5.107)	0.155
Social disability	1/102 (1.0)	10/198 (5.1)	5.352 (0.742–235.415)	0.131
Handicap	0/102 (0.0)	8/198 (4.0)	Not estimable	0.069
Overall impact	18/102 (17.6)	50/198 (25.3)	1.574 (0.838–3.062)	0.176

Values are n/N (%). ORs compare the second group with the first. No missing data. OR, odds ratio; CI, confidence interval. Exact two-sided *p* values are shown. ‘Not estimable’ indicates a zero cell.

**Table 7:** Exploratory unadjusted associations between reason for dental attendance and OHIP-14 outcomes, 2024.

OHIP-14 Outcome	Routine/Prevention (n = 212) n/N (%)	Urgent Care (n = 88) n/N (%)	Unadjusted OR (95% CI)	*p* Value
Functional limitation	6/212 (2.8)	6/88 (6.8)	2.503 (0.649–9.662)	0.206
Physical pain	16/212 (7.5)	21/88 (23.9)	3.819 (1.783–8.334)	<0.001
Psychological discomfort	17/212 (8.0)	22/88 (25.0)	3.803 (1.806–8.142)	<0.001
Physical disability	3/212 (1.4)	10/88 (11.4)	8.857 (2.205–51.378)	<0.001
Psychological disability	18/212 (8.5)	18/88 (20.5)	2.761 (1.276–5.983)	0.009
Social disability	5/212 (2.4)	6/88 (6.8)	3.016 (0.744–12.861)	0.134
Handicap	1/212 (0.5)	7/88 (8.0)	18.053 (2.263–825.556)	0.002
Overall impact	32/212 (15.1)	36/88 (40.9)	3.874 (2.119–7.137)	<0.001

Values are n/N (%). ORs compare the second group with the first. No missing data. OR, odds ratio; CI, confidence interval. Exact two-sided *p* values are shown. ‘Not estimable’ indicates a zero cell.

## Data Availability

No new data were created or analyzed in this study. The individual-level dataset is not publicly available because of participant confidentiality and the risk of re-identification in a small, culturally identifiable rural community. Requests for de-identified data, the data dictionary, or analysis documentation may be directed to the corresponding author and will be considered subject to applicable ethical approval and privacy safeguards.
